# Estrogen promotes progression of hormone-dependent breast cancer through CCL2-CCR2 axis by upregulation of Twist via PI3K/AKT/NF-κB signaling

**DOI:** 10.1038/s41598-018-27810-6

**Published:** 2018-06-22

**Authors:** Rui Han, Shanzhi Gu, Yujiao Zhang, Anqi Luo, Xin Jing, Lin Zhao, Xinhan Zhao, Lingxiao Zhang

**Affiliations:** 1 Department of Oncology, the First Affiliated Hospital of Medical School of Xi’an Jiaotong University, 277 West Yanta Road, Xi’an, Shaanxi Province 710061 China; 2 Department of Forensic Medicine, Medical School of Xi’an Jiaotong University, 76 West Yanta Road, Xi’an, Shaanxi Province 710061 China

## Abstract

The chemokine (C-C motif) ligand 2 (CCL2) with its cognate receptor chemokine (C-C motif) receptor 2 (CCR2) plays important roles in tumor invasion and metastasis. However, the mechanisms and mediators for autocrine CCL2 and CCL2-CCR2 axis remain elusive in breast cancer. Here we examined the levels of CCL2 in 4 breast cancer cell lines along with 57 human breast cancer specimens and found them significantly increased with presence of 17β-estradiol (E2) in estrogen receptor (ER)-positive breast cancer cells, while anti-estrogen treatment weakened this enhancement. CCL2 expression positively correlated with Twist staining and aggressiveness of breast cancer. Estrogen exposure facilitated the proliferation, invasion and metastasis of hormone-dependent breast cancer and promoted angiogenesis via the increased secretion of CCL2 *in vitro* and *in vivo*, which could be suppressed by disruption of CCL2-CCR2 axis with CCR2 antagonist RS102895. Knockdown of Twist in MCF-7 cells significantly inhibited E2-induced CCL2 production, indicating an essential role of Twist in CCL2 regulation under estrogenic condition. Our data show the hormonal regulation on CCL2-CCR2 axis is associated with enhanced Twist expression via activation of ERα and PI3K/AKT/NF-κB signaling. Thus, CCL2-CCR2 axis may represent as a novel therapeutic target eagerly needed for hormone-dependent breast cancer.

## Introduction

Chemokines are a family of cytokines with small molecular mass and chemotactic ability. The complex chemokine-receptor signaling in cancer leads to the tumor growth, invasion, and metastasis to specific sites^1^. CCL2, also known as monocyte chemotactic protein-1(MCP-1), is a primary chemoattractant for T lymphocytes, monocytes, macrophages, mast cells and endothelial cells^2–4^. CCL2 is highly expressed by various breast cancer cells such as 4T1, along with both the hematopoietic and non-hematopoietic cells like macrophages in tumor stroma^5^. CCL2 exhibits a particular affinity for CCR2, which is expressed in multiple tissues, including blood, brain, liver, lung, colon, ovary, pancreas, kidney and spleen^6–9^, and their binding activates a series of downstream biological effects in breast cancer^10,11^. CCL2 has been shown to play crucial roles in breast and prostate tumor progression by facilitating cancer cell proliferation and survival, recruiting macrophages, and inducing angiogenesis through multiple mechanisms, including increasing survivin expression by activation of PI3K/AKT pathway and direct induction of vascular endothelial growth factor-A (VEGF-A) through activation of p42/44 mitogen-activated protein kinase (MAPK)^12–15^. A total blockade of CCL2 both from the tumor and the stroma decreased recruitment of CCR2+ monocytes, inhibited tumor metastasis and extended survival time of tumor-bearing mice^10,16^. Despite of those experimental studies that strongly support CCL2 as a pro-tumorigenic role in breast cancer, therapeutics aimed at interfering CCL2-CCR2 axis have turned out to be disappointing in the clinical trials^17–19^. Therefore, understanding the mechanisms of CCL2 regulation and CCL2-CCR2 signaling could shed new light on how to make CCL2 or CCR2 blockade more resultful.

Estrogen, one of the high risk factors for breast cancer, binds to ER to activate signaling pathways and exert biological properties such as stimulating cell proliferation and inhibiting apoptosis^20^. Blocking the action of estrogen with selective estrogen receptor modulator (SERM) and aromatase inhibitor (AI) is a huge improvement in current breast cancer therapy^21^. However, adjuvant tamoxifen treatment for 5 to 10 years has exhibited a relatively high risk of recurrence and its side effects are severe^22^. Hence, estrogen-regulated signaling pathways in ER+ breast cancer need to be further explored to identify new drug targets.

The transcription factor Twist1 (Twist), has been shown to induce epithelial-mesenchymal transition (EMT) and play a crucial role in tumor metastasis and multidrug resistance^23–25^. In human breast tumors, Twist is usually over-expressed, which is majorly associated with distant metastasis, high degree of malignancy and unfavorable prognosis^26,27^. In tumor xenograft models, Twist is indicated to promote angiogenesis^28^, which is one critical contributor to tumor metastasis. However, the relevance of Twist expression with CCL2 modulation in human breast cancer still remains unclear.

In the current study, we tested the levels of CCL2 under estradiol exposure in different breast cancer cells, and investigated the roles of CCL2-CCR2 axis in estrogen-induced biological effects *in vitro* and *in vivo*. Subsequently, we employed lower Twist-expressing MCF-7 cells to examine the influence of modulating Twist expression on the E2-induced autocrine CCL2. Furthermore, we explored the underlying mechanisms of estrogen-triggered CCL2 regulation in ER+ breast cancer cells. Our data demonstrate that estrogen may positively regulate CCL2 synthesis and autocrine by upregulation of Twist through ERα activation and PI3K/AKT/NF-κB signaling instead of MEK/ERK pathway. Taken together, CCL2-CCR2 axis is a novel therapeutic target for hormone-dependent breast cancer.

## Results

### The expression of CCL2 varies under estrogen exposure in different breast cancer cell lines and correlates with aggressiveness of breast cancer

**Figure 1:**
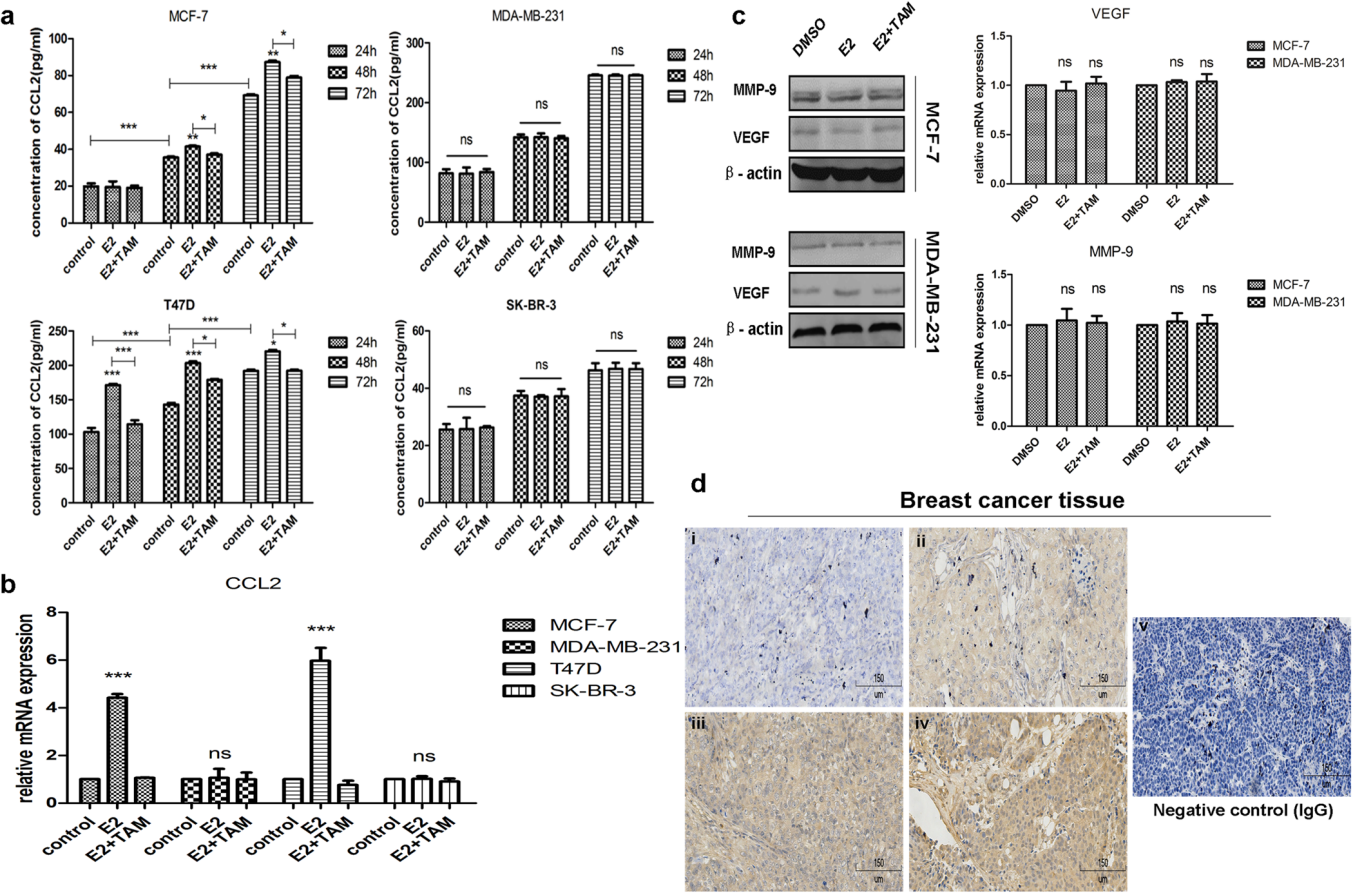
Expression of CCL2 in human breast cancer cell lines and tissues. **(a)** Conditioned media at the absence or presence of estrogen from 4 cell lines were collected and CCL2 levels were determined by ELISA; **(b)** mRNA expression of CCL2 in 4 cell lines at the absence or presence of estrogen were detected by qRT-PCR; **(c)** the mRNA and protein levels of VEGF and MMP-9 in MCF-7 and MDA-MB-231 cells at the absence or presence of estrogen were measured by qRT-PCR and Western blot. Cropped images are from those samples and antibodies processed and run on different gels. Full-length blots for cropped gels are shown in Supplementary Fig. S4a–b; **(d)** Representative images of anti-CCL2 staining detected by IHC in human breast tumour tissues. (i) Invasive ductal carcinoma, Stage I; (ii) Invasive ductal carcinoma, Stage II; (iii) Invasive ductal carcinoma, Stage III; (iv) Invasive ductal carcinoma, Stage III; (v) Negative IgG control. Bars: 150 μm. Data are representative images or expressed as the mean ± SEM of each group of cell lines from three independent experiments. *P < 0.05, **P < 0.01, ***P < 0.001 vs control or DMSO; ns, not significant.

First, we examined CCL2 expression in the culture media of different breast cancer cell lines, including MCF-7, T47D, MDA-MB-231 and SK-BR-3. As demonstrated in Fig. 1a–b, ER+ MCF-7 and T47D cells showed higher protein and mRNA levels of CCL2 with the presence of E2 and tamoxifen could partially attenuate the effects, while no statistical significance was found among those groups in ER-negative MDA-MB-231 or SK-BR-3 cells. In terms of other proteins secreted in the culture media such as VEGF and MMP-9, their mRNA and protein levels showed no significant changes in either MCF-7 or MDA-MB-231 cells (Fig. 1c). IHC staining results showed positive signals of CCL2 were primarily located in the cytoplasm of tumor cells (Fig. 1d). 36 (63.2%) out of the 57 cases were positive for CCL2. As shown in Table 1, CCL2 expression was markedly correlated with tumor status (*P* = 0.004), lymph node metastasis (*P* = 0.007) and TNM stage (*P* = 0.026). However, there was no significant relationship of CCL2 expression with ER status (*P* = 0.263), PR status (*P* = 0.270), Her-2 expression (*P* = 0.159) or other clinicopathological features.

**Table 1:** Relationship between the clinicopathological variables and CCL2 expression.

Characteristic	Case	CCL2 expression		*χ* ^2^	*P* value^1^
		Negative	Positive		
Age					
≤50	27	7	20	2.627	0.169
>50	30	14	16		
Menses status					
Premenopause	28	9	19	0.522	0.585
Menopause	29	12	17		
TNM stage^2^					
I–II	35	17	18	5.362	0.026^*^
III–IV	22	4	18		
Tumor status^2^					
T1	23	14	9	9.567	0.004^*^
T2–T4	34	7	27		
Lymph nodal status^2^					
N0	27	15	12	7.721	0.007^*^
N1–N3	30	6	24		
ER status					
Negative	23	6	17	1.917	0.263
Positive	34	15	19		
PR status					
Negative	31	9	22	1.781	0.270
Positive	26	12	14		
Her-2					
Negative	36	16	20	2.427	0.159
Positive	21	5	16		

Data are real case numbers. ^1^*P* values were determined by two-tailed Chi-square test or Fisher’s exact test. ^2^The TNM stage, tumor status, and lymph nodal status were classified according to the international standards for staging breast cancer.

^*^Statistically significant.

### Estrogen exposure promotes ER+ breast cancer cell proliferation, migration and invasion via the upregulation of autocrine CCL2

**Figure 2:**
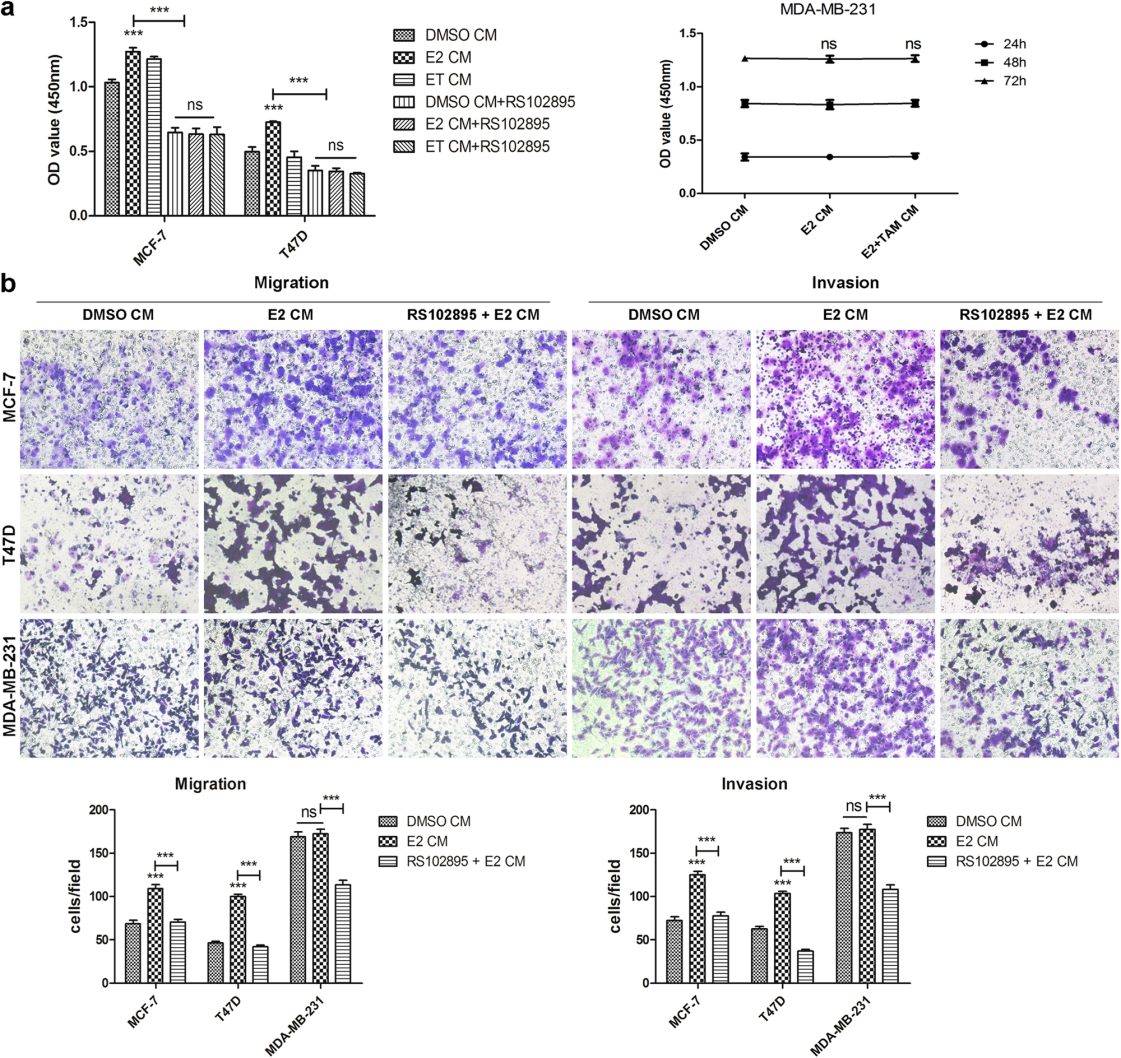
Estrogen exposure promotes ER+ breast cancer cell proliferation, migration and invasion via upregulation of autocrine CCL2. **(a)** MCF-7, T47D and MDA-MB-231 cells were supplied with their own DMSO-treated, E2-treated or ET(E2 plus tamoxifen)-treated CM for 24 h, 48 h or 72 h and then detected for cell viability by WST-8 assay. **(b)** 3 cell lines were supplied with or without E2-treated CM in the pretreatment or absence of RS102895 (20 μM). Then their migratory ability at 24 h and invasive ability at 36 h post incubation were respectively examined by migration and invasion assay. Data are representative images or presented as the mean ± SEM of different groups from three independent experiments. ***P < 0.001 vs control or DMSO CM; ns, not significant.

Since we have found E2 could directly increase CCL2 expression in ER+ breast cancer cells, and CCL2-CCR2 axis also coordinated breast cancer cell viability, migration and invasion *in vitro* as shown in Supplementary Fig. S1, which was consistent with a previous study reported by Fang W. B. *et al*.^11^, further experiments were conducted to verify how estrogen exposure affected biological functions of breast cancer cell and whether CCL2-CCR2 axis involved in it. First, WST-8 assay results shown in Fig. 2a demonstrated that E2-treated CM from ER+ cells were able to promote cell proliferation and this effect could be reversed by RS102895, a potent and specific CCR2 antagonist, which blocks CCL2 signaling through CCR2 by occupation of a binding site on the extracellular side of this receptor^29^. In order to determine whether hormone receptor (HR) status is essential for the observed effects, HR negative MDA-MB-231 cells were also examined. However, E2-treated CM from this cell line did not affect its cell viability (Fig. 2a). As shown in Fig. 2b, the number of migrated and invaded cancer cells mediated by CM generated from E2-treated ER+ cells all increased immensely and pretreatment with RS102895 reduced these numbers strikingly. No significant changes were observed in either migration or invasion of HR negative MDA-MB-231 cells (Fig. 2b). Hence, estrogen exposure exhibits stronger ability at promoting cell viability, migration and invasion, which is related to HR status of breast cancer cells.

### Estrogenic condition regulates HUVEC viability, motility and tube formation ability via CCL2-CCR2 axis *in vitro*

**Figure 3:**
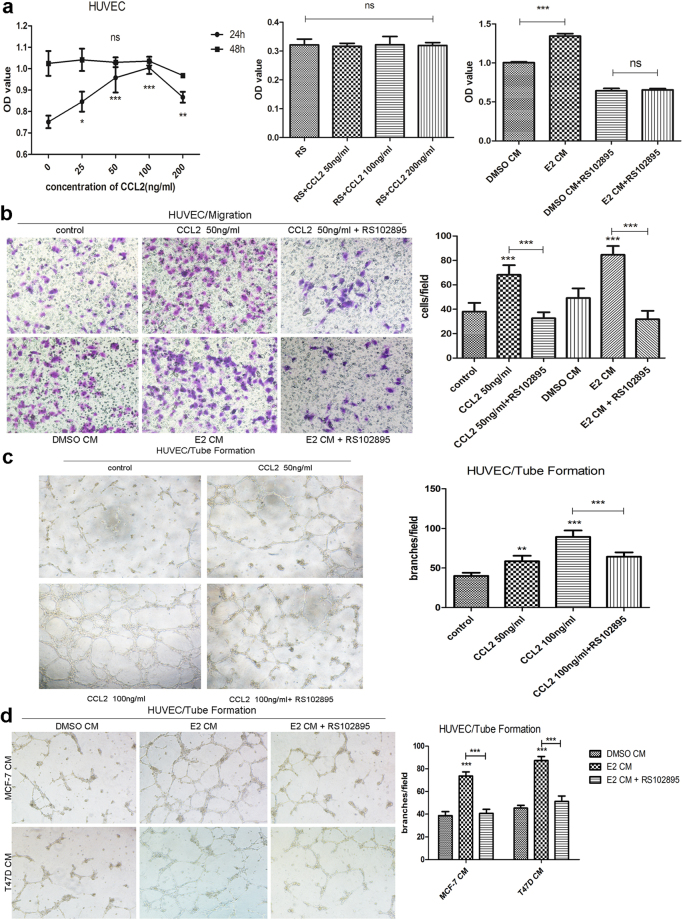
Estrogenic condition regulates HUVEC viability, motility and tube formation via CCL2-CCR2 axis *in vitro*. **(a)** The proliferation of HUVECs that were supplied with or without different concentrations of rhCCL2 or DMSO/E2-treated CM in the present or absence of RS102895 (20 μM) at the indicated time was measured by WST-8 assay; **(b)** HUVECs were supplied with or without 50 ng/ml rhCCL2 or DMSO/E2-treated CM from MCF-7 and T47D cells in the pretreatment or absence of RS102895 (20 μM) and their migratory ability at 24 h post incubation was determined by migration assay; **(c)** HUVECs were treated with or without various concentrations of rhCCL2 in the pretreatment or absence of RS102895 (20 μM) and their ability to form capillary-like tubes were measured using tube formation assay. **(d)** HUVECs were incubated in the presence of DMSO-treated or E2-treated CM from MCF-7 and T47D cells with the pretreatment of RS102895 or not. Then HUVECs were captured 10 hours after CM was added. Images are representative and data are shown as the mean ± SEM from three separate experiments. *P < 0.05, **P < 0.01, ***P < 0.001 vs. control or DMSO CM; ns, not significant.

The ability of tumor cells to promote vascularization, or blood vessel growth, plays crucial roles in many steps of tumor development and progression. In order to determine whether breast cancer cells under estrogenic condition can promote angiogenesis, a series of further experiments were conducted. First, we examined if CCL2, the factor secreted more by HR+ breast cancer cells at the presence of estradiol as we demonstrated in Fig. 1, was able to attract endothelial cells or stimulate their proliferation. Then we investigated how estrogen exposure exhibits those angiogenic effects by using conditioned media from HR+ cells and CCR2 antagonist RS102895. In WST-8 assay, HUVECs were treated with a series of concentrations of rhCCL2 (0, 25, 50, 100 and 200 ng/ml) for 24 or 48 h and examined for cell viability. When HUVECs were supplied with rhCCL2 for 24 h, cell viability was enhanced and there was a peak at the concentration of 100 ng/ml. However, no significant changes were observed at 48 h despite of the increasing concentration (Fig. 3a). A similar pattern was showed with the presence of RS102895 at 24 h (Fig. 3a). Additionally, E2-treated CM from MCF-7 cells was able to promote HUVECs proliferation and RS102895 neutralized this impact (Fig. 3a). Meanwhile cell migration assay results revealed that both CCL2 alone and CM from E2-treated ER+ cells could enhance the migratory ability of HUVECs, which was also abrogated with pretreatment of RS102895 (Fig. 3b). To investigate if estrogen exposure impacts the secretion of angiogenic proteins and whether CCL2-CCR2 axis involved in the process, tube formation assays were implemented. *In vitro* studies has demonstrated that in the presence of angiogenic factors such as VEGF and chemokines like CCL2, endothelial cells proliferate and form tube structures resembling capillaries when plated on a reconstituted basement membrane^30,31^. As shown in Fig. 3c, both 50 ng/ml and 100 ng/ml rhCCL2 increased tube formation ability of HUVECs compared with the absence of CCL2. Moreover, presence of 100 ng/ml rhCCL2 with the pretreatment of RS102895 reduced almost a third of HUVEC branches compared to the presence of 100 ng/ml rhCCL2 alone. HUVECs that were cultured with CM from E2-treated ER+ cells generated nearly two-fold more branches compared to those incubated with CM collected from cells without estrogen exposure (Fig. 3d). Similarly, HUVECs pretreated with RS102895 then incubated with E2-treated CM, had fewer branches than incubated with E2-treated CM alone (Fig. 3d). These results suggest that estrogenic condition alters HUVEC viability, motility and tube formation ability by increasing the secretion of pro-angiogenic factor CCL2 via CCL2-CCR2 axis.

### Knockdown of Twist in MCF-7 cells significantly inhibits E2-induced production of CCL2

**Figure 4:**
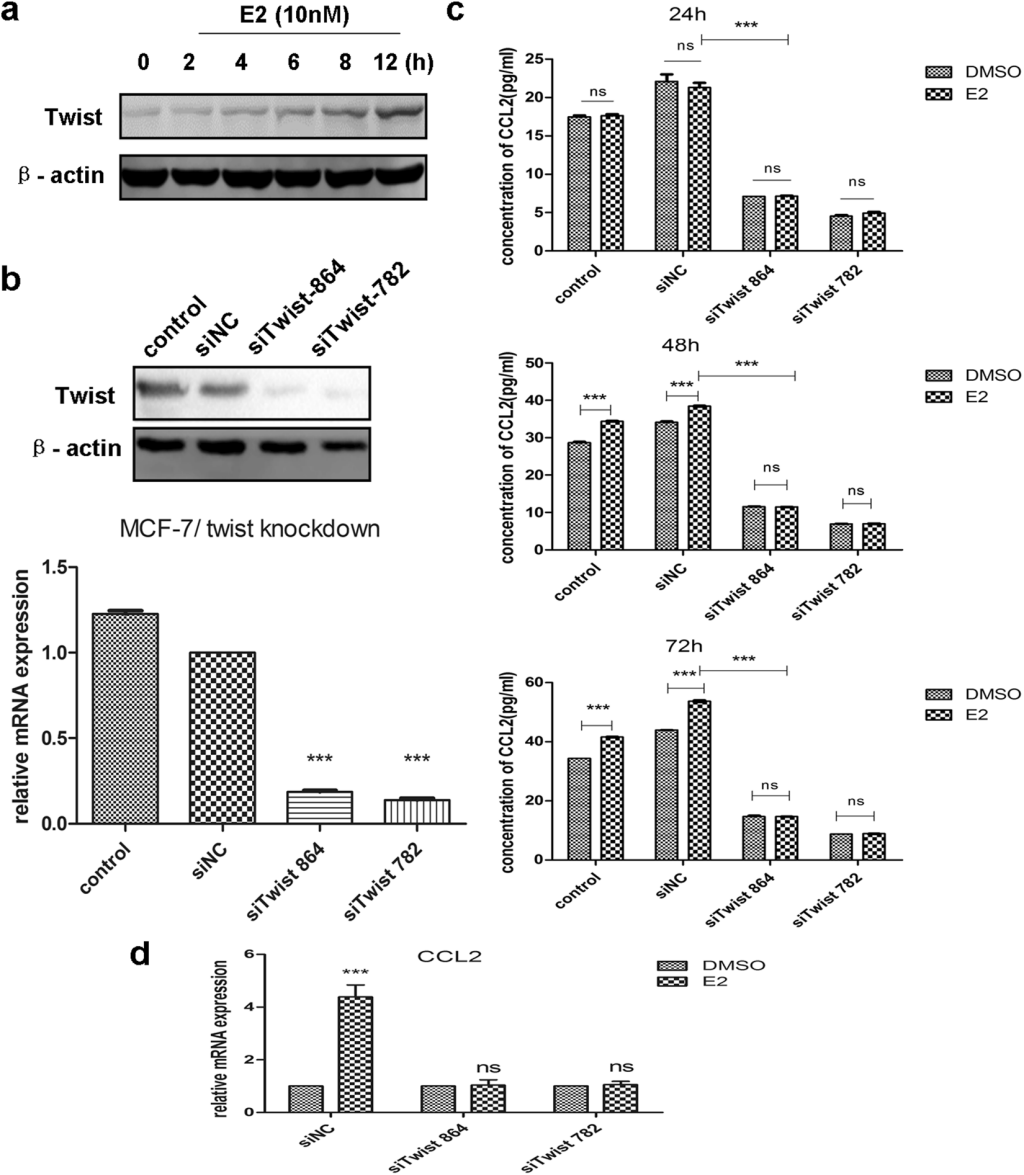
Knockdown of Twist in MCF-7 cells significantly inhibits E2-induced production of CCL2. **(a)** The protein levels of Twist at the indicated time points in MCF-7 cells cultured with 10 nM E2 were determined by Western blot. Cropped images are from samples run on the same gel. Full-length blots are displayed in Supplementary Fig. S4b; **(b)** The relative levels of Twist expression in MCF-7 cells after siRNA transfection were measured by qRT-PCR and Western blot. Full-length blots are demonstrated in Supplementary Fig. S4c; **(c)** The levels of E2-induced CCL2 secretion in MCF-7 cells at the indicated time points after knockdown of Twist were detected by ELISA analysis; **(d)** The mRNA levels of E2-induced CCL2 in MCF-7 cells after knockdown of Twist was determined by qRT-PCR. Data are shown as mean ± SEM and images are representative from three independent experiments. ***P < 0.001 vs. the controls; ns, not significant.

In HR+ breast cancer, estrogen exposure exerts direct and indirect effects in promoting tumor growth and invasion via tumor-derived CCL2. However, the mechanisms for E2-triggered CCL2 regulation are not well defined yet. Transcription factor Twist has been shown to associate with tumor survival and progression and to induce angiogenesis at the presence of CCL2 and macrophage recruitment^32^, suggesting that Twist may get involved with CCL2 regulation. Interestingly, our Western blot results revealed E2 could increase Twist protein level of MCF-7 cells in a time-dependent manner (Fig. 4a). On the basis of this knowledge, we focused our further studies on examining whether Twist participates in E2-induced CCL2 modulation. First, to clarify the relationship between Twist and CCL2 expression in breast cancer, we detected their presence by IHC in 57 human breast tumor specimens. Statistical analysis showed that expression of CCL2 positively correlated with Twist staining (R = 0.438, *P* < 0.001) (Table 2). Hence, we transfected MCF-7 cells with different Twist specific siRNAs to investigate if silenced Twist expression could affect CCL2 production *in vitro*. The knockdown efficiency of Twist expression levels were examined by qRT-PCR and Western blot (Fig. 4b). Then the effect of altered Twist expression on CCL2 levels was determined. Compared with the controls, knockdown of Twist in MCF-7 cells significantly inhibited CCL2 secretion (*P* < 0.001, Fig. 4c). More importantly, it abrogated E2-induced autocrine CCL2 at the indicated time points (Fig. 4c). The mRNA expression of CCL2 enhanced by E2 was counteracted in different MCF-7/siTwist cells too (Fig. 4d). These data indicates Twist may be crucial at regulating CCL2 production in ER+ breast cancer. Hence, further experiments were conducted to investigate the mechanisms underlying how estrogen affects CCL2 expression.

**Table 2:** Correlation between CCL2 and Twist expression in human breast tumor specimens.

Twist expression	Case	CCL2 expression				R	*P* value
		−	1+	2+	3+		
−/1+	13	10	2	1	0		
2+	27	7	6	12	2	0.438	0.001^*^
3+	17	4	1	7	5		

^*^Statistically significant.

### Estrogen enhances Twist expression via activation of AKT and NF-κB phosphorylation in ER+ breast cancer cells

**Figure 5:**
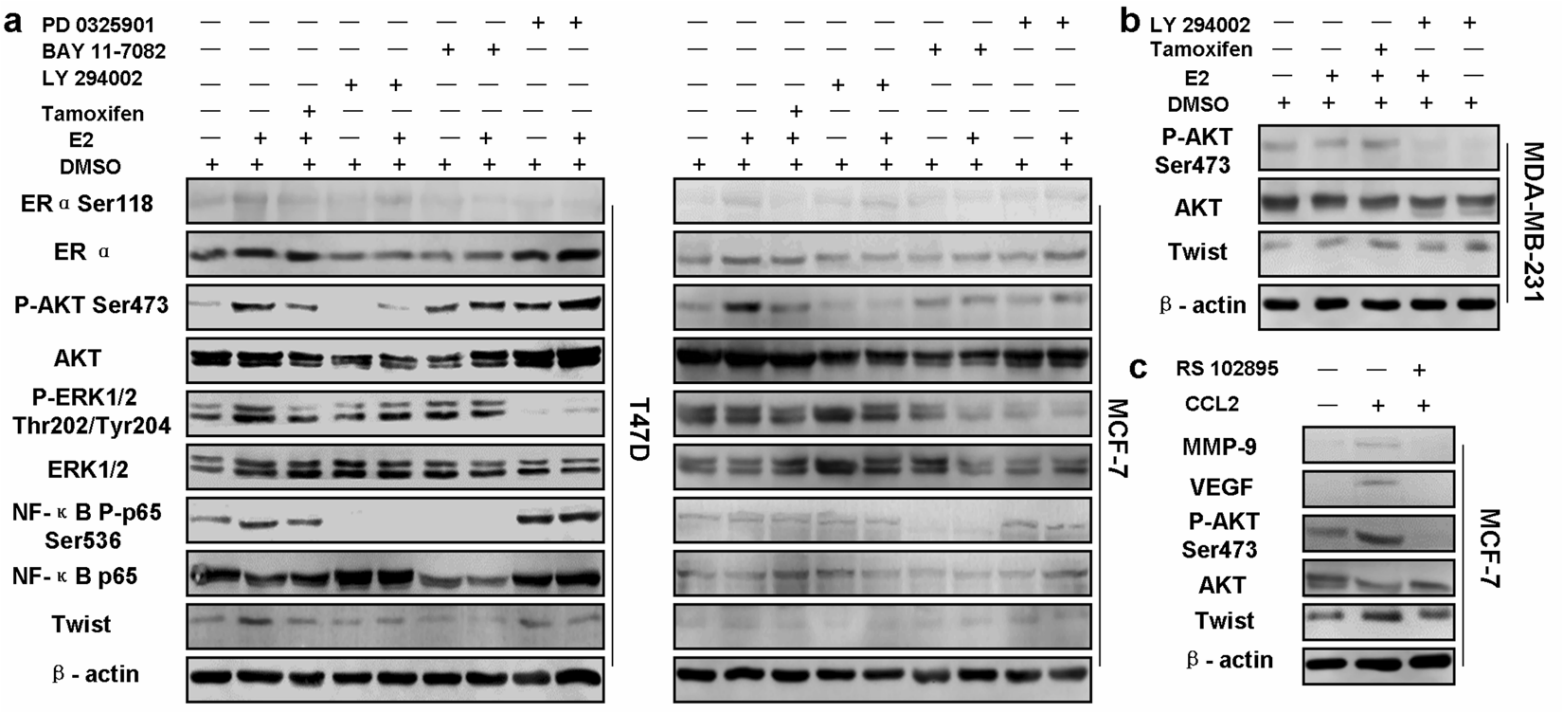
Estrogen enhances Twist expression via activation of AKT and NF-κB phosphorylation in ER+ breast cancer cells. **(a)** T47D and MCF-7 cells were pretreated with 0.5 μM Tamoxifen, 50 μM PI3K inhibitor LY294002, 10 μM IKK inhibitor Bay11–7082 or 10 μM MEK inhibitor PD 0325901 for 12 h, followed by stimulation of 10 nM E2 for 6 h or not. The protein levels of ERα, AKT, ERK1/2, NF-κB p65 and Twist as well as ERα, AKT, ERK1/2 and NF-κB p65 phosphorylation in different groups of T47D and MCF-7 cells with indicated treatments were measured by Western blot. **(b)** MDA-MB-231 cells were pretreated with 0.5 μM Tamoxifen and 50 μM LY294002 for 12 h, followed by stimulation of 10 nM E2 for 6 h or not. The protein levels of AKT and Twist as well as AKT phosphorylation on Ser473 were then detected by Western blot. **(c)** MCF-7 cells were pretreated with 20 μM RS102895 for 12 h, followed by stimulation of 20 ng/ml rhCCL2 for 24 h. The protein levels of MMP-9, VEGF, AKT and Twist as well as AKT phosphorylation were then detected by Western blot. Images are shown representatively from 3 independent experiments. Cropped images are from those samples and antibodies processed and run on different parts of gels. Full-length blots are displayed in Supplementary Fig. S4b.

**Figure 6:**
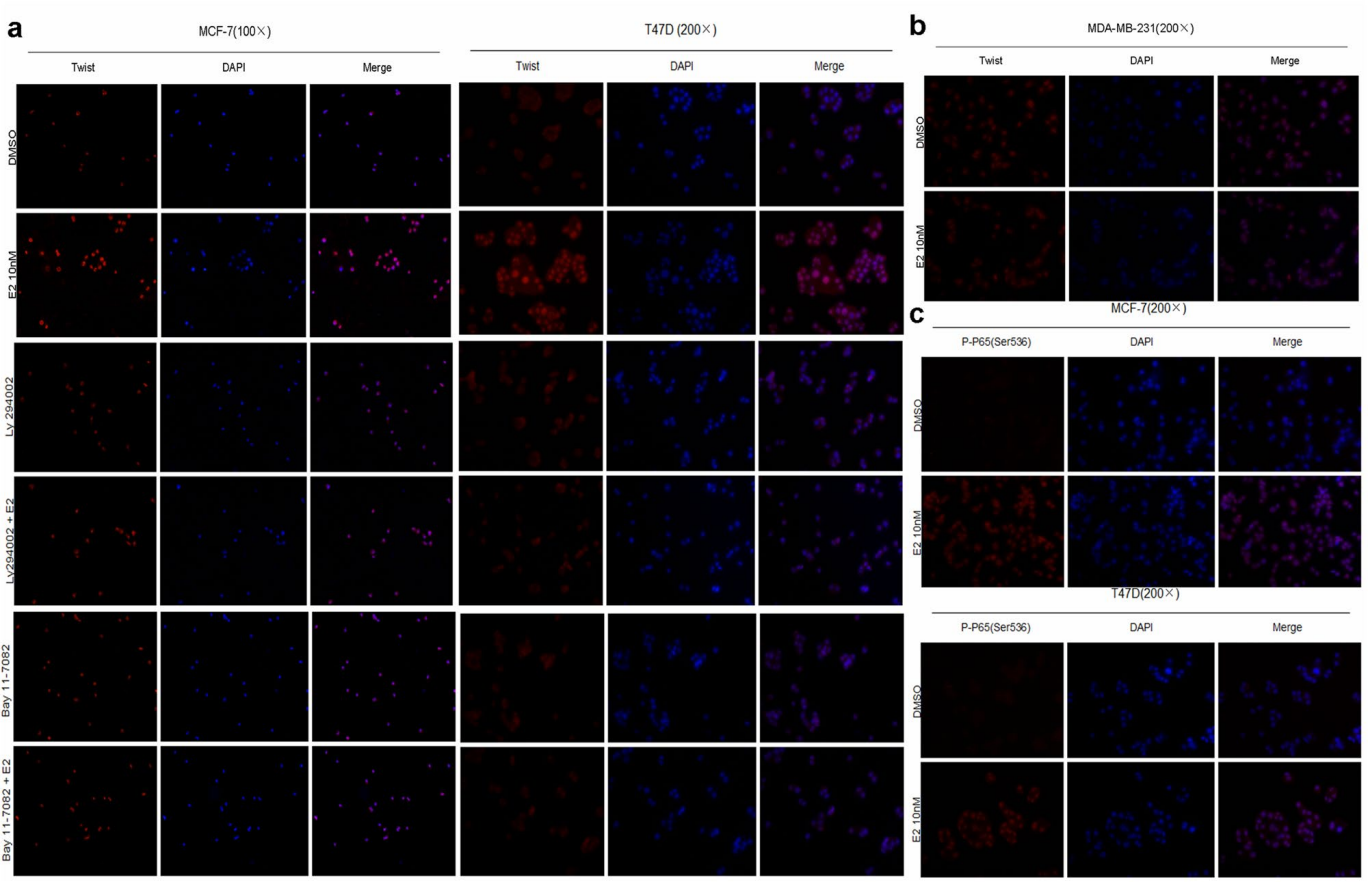
Estrogen enhances nuclear translocation of Twist via activation of NF-κB p65 phosphorylation in ER+ breast cancer cells. **(a)** MCF-7 and T47D cells were pretreated with 50 μM LY294002 or 10 μM Bay11–7082 for 12 h, followed by stimulation with 10 nM E2 for 6 h or not. **(b)** MDA-MB-231 cells were incubated for 6 h with or without 10 nM E2. **(c)** MCF-7 and T47D cells were cultured in the presence or absence of 10 nM E2 for 6 h. Immunofluorescence assays were performed with anti-Twist and anti-NF-κBp-p65. Images are shown representatively from 3 independent experiments.

Previously several studies have indicated that both NF-κB and Twist are involved in tumor invasion and metastasis^33,34^. To investigate the underlying molecular mechanisms for regulation of CCL2 by estrogen, the protein levels of ERα, AKT, ERK1/2, NF-κB p65 and Twist as well as ERα, AKT, ERK1/2 and NF-κB p65 phosphorylation in different cells with indicated treatments were measured by Western blot. As demonstrated in Fig. 5a, enhanced levels of AKT and NF-κB p65 phosphorylation and increased Twist expression were shown in both MCF-7 and T47D cells incubated with E2 than those without it. However, there were no significant changes of ERK1/2 expression in two cell lines between E2 and the control group (Fig. 5a). Treatment with MEK inhibitor PD0325901 didn’t reduced the levels of NF-κB p65 phosphorylation or Twist expression (Fig. 5a). Intriguingly, treatment with E2 also slightly increased ERα phosphorylation in both ER+ cells (Fig. 5a) while the same treatment didn’t significantly change the AKT phosphorylation or Twist expression in ER-negative cells (Fig. 5b). More interestingly, induction of rhCCL2 significantly enhanced the AKT phosphorylation and Twist expression in MCF-7 cells, while pretreatment with RS102895 abrogated the effects (Fig. 5c), strongly indicating an essential role of Twist in autocrine CCL2. In addition, there was also a similar pattern of MMP-9 and VEGF expression observed in MCF-7 cells (Fig. 5c). As illustrated in Fig. 6a,c, E2 enhanced nuclear translocation of Twist and phosphorylated NF-κB p65 in both ER+ cells, while the enhancement of Twist nuclear translocation was not found in MDA-MB-231 cells (Fig. 6b). Overall, the data indicate that in HR-positive breast cancer, hormonal regulation on CCL2 expression might be strongly related to modulation of Twist expression via PI3K/AKT/NF-κB signaling instead of MEK/ERK pathway.

### Estrogen promotes the growth and metastasis of implanted ER+ breast tumors via CCL2-CCR2 signaling in both autocrine and paracrine manners in mice

**Figure 7:**
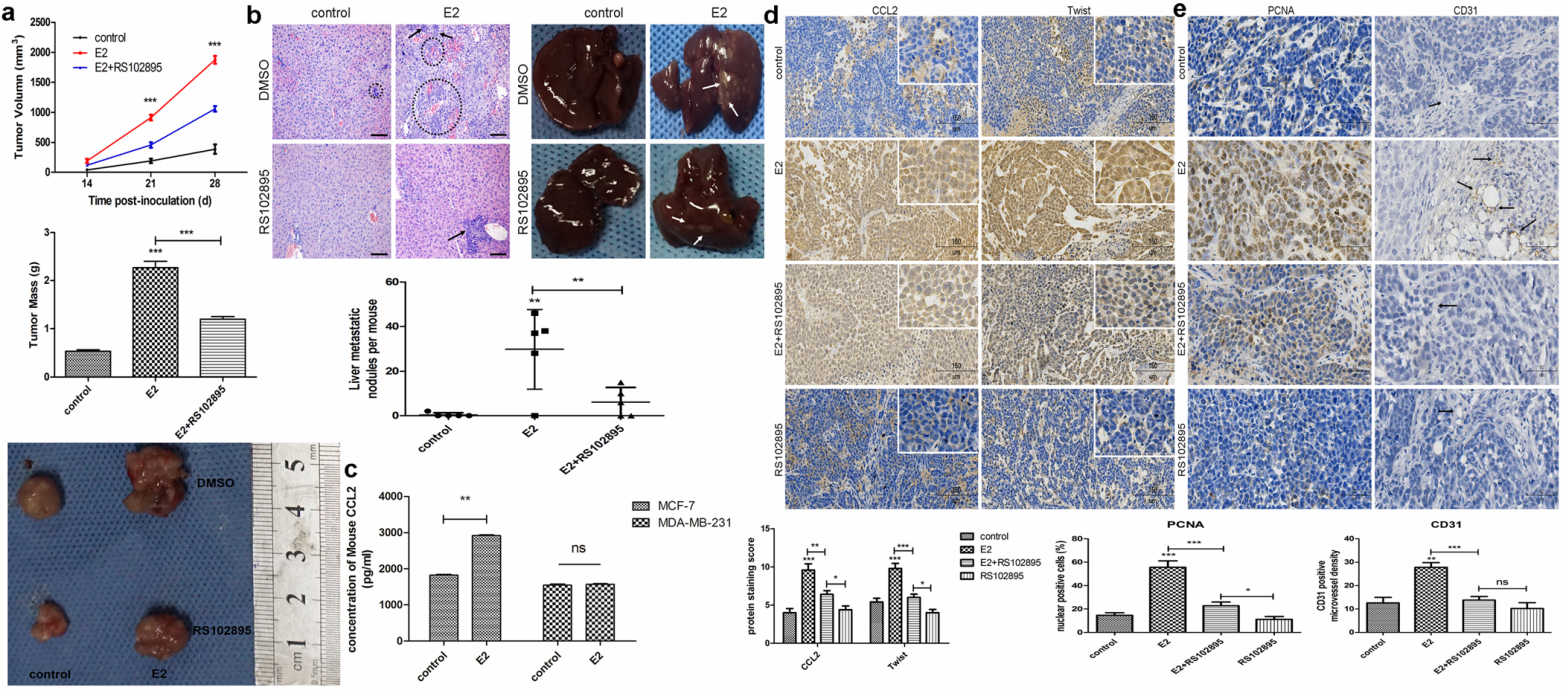
Estrogen promotes the growth and metastasis of implanted HR+ breast tumors via CCL2-CCR2 axis in both autocrine and paracrine manners *in vivo*. **(a)** The volumn and mass of inoculated MCF-7 breast tumors; **(b)** The liver metastatic tumors generated from MCF-7 cells were observed with macrography and counted. The micrometastasis in the liver tissue sections were stained by H&E (scale bars: 100 μm); arrows indicate the metastatic nodules. The numbers of metastatic tumors on liver were quantitatively analyzed. **(c)** The CCL2 levels in serum from different groups of mice inoculated with MCF-7 cells or MDA-MB-231 cells were detected by ELISA analysis. **(d)** and **(e)** The CCL2, Twist, PCNA and CD31 expression in the MCF-7 tumors were shown by IHC staining. Insets show magnification for CCL2 and Twist staining detail; arrows indicate the positively CD31 staining. Images are representative and data are expressed as the mean ± SEM of 3 independent experiments. *P < 0.05, **P < 0.01, ***P < 0.001 vs. the controls; ns, not significant.

Lastly, the effect of estrogen on implanted breast tumor and its underlying mechanism *in vivo* was analyzed. The volumes and mass of the E2-treated tumors were strikingly larger than the control tumors while RS102895 could moderately attenuate the E2-induced tumor growth (Fig. 7a). Although only one from the control group of mice generated a few metastatic nodules on liver, much more nodules were observed in four out of five mice in the E2-treated group. Moreover, some metastatic nodules on liver also appeared in two mice with E2/RS102895-treated tumors (Fig. 7b). Quantitative analysis showed that the mean numbers of these nodules on liver from E2-treated mice were significantly more than that from the control mice and E2/RS102895-treated mice (*P* < 0.01, Fig. 7b). Histological staining clearly figured the liver metastatic tumor from different groups of mice (Fig. 7b). ELISA analysis revealed the CCL2 levels in serum from E2-treated mice inoculated with MCF-7 cells were higher than the control mice (*P* < 0.01, Fig. 7c), while no significant difference was found between these groups of mice inoculated with MDA-MB-231 cells (ns, Fig. 7c). IHC analysis showed the intensity of anti-CCL2 and anti-Twist staining in the E2-treated group of tumors was much stronger than that in the control group and E2/RS102895 group (*P* < 0.001, Fig. 7d). Moreover, the intensity of CCL2 and Twist staining in the tumors from E2/RS102895-treated mice was also stronger than those from mice treated with RS102895 alone (*P* < 0.05, Fig. 7d). Negative IgG controls were displayed in Supplementary Fig. S2. Similarly, percentage of tumor cells with anti-PCNA staining positively in nuclear from the E2-treated group was significantly higher than that from the control and E2/RS102895 group, and this percentage of E2/RS102895-treated tumors was also higher than that of RS102895 group (*P* < 0.001 and *P* < 0.05 separately, Fig. 7e). These findings suggest the possibility that CCL2 might largely contribute to E2-induced tumor growth through a direct effect on cancer cells. Considering vascular formation could also be affected by CCL2-CCR2 axis *in vitro*, the vascular density in the E2-treated and E2/RS102895-treated tumors was analyzed using anti-human CD31 IHC staining. The microvessel density of E2-treated tumors was more than that of the control and E2/RS102895-treated tumors (*P* < 0.01 and *P* < 0.001 separately, Fig. 7e). Interestingly, no significant difference of anti-CD31 staining was observed between tumors from E2/RS102895 and RS102895 group (ns, Fig. 7e). Similar patterns were also shown in IHC staining for mature macrophage marker F4/80 (Supplementary Fig. S3), suggesting an essential role of CCL2-CCR2 signaling in E2-induced vascularization and macrophage infiltration in tumor stroma. Therefore, estrogen could promote tumor growth and liver metastasis via CCL2-CCR2 axis which acts in both autocrine and paracrine manners in HR+ xenograft tumor models.

## Discussion

Exposure to estrogen is a crucial factor for progression and metastasis in patients with breast cancer^35^. Anti-estrogen therapeutic regimes help lower the recurrence and mortality risk but some side effects of this traditional treatment are long-term and severe such as thromboembolic events, osteoporosis, fracture, endometrial cancer and sexual dysfunction^22,36^. Hence, novel hormone-dependent therapeutic targets are urgently needed for improved treatment of breast cancer. Moreover, reduction of endogenous estrogen exposure might make traditional tamoxifen therapy or other treatments work better. Our findings indicate the critical roles of CCL2-CCR2 axis in the proliferative and metastatic properties of breast cancer cells, which is consistent with several previous studies^10,11^. Despite CCL2 being implicated as a prominent contributor in tumor progression, the molecular mechanisms of CCL2 induction still remain largely unknown. A recent study reported by Nakatsumi *et al*. showed that insulin-induced mTORC1 activation could regulate CCL2 expression in a manner independent of NF-κB signaling by dephosphorylating the transcription factor FOXK1 in HeLa cells^37^. Here we correlate CCL2 expression to transcription factor Twist using human breast tumor specimens and for the first time identify a mechanism through which estrogenic condition facilitates CCL2 synthesis to promote metastasis and angiogenesis in HR+ breast cancer, to our best knowledge.

Our results contradict with Fanti *et al*. who reported a downregulatory effect of estrogen on CCL2 expression in murine mammary tissue^38^. Instead, we observed that E2 significantly increased the levels of CCL2 in ER+ MCF-7 and T47D cells as well as serum from nude mice bearing ER+ implanted breast tumors. Our findings aren’t in line with Seeger *et al*., who reported E2 (0.1 nM) alone elicited no effect on CCL2 synthesis of MCF-7 cells^39^, which might due to the different concentration used for estradiol. Moreover, they did observe that in combination with TNF-α, E2 significantly induced a further stimulation of CCL2 by about 20%, and anti-estrogen tamoxifen was able to partly inhibit the action of TNF-a and estradiol, suggesting E2-induced effect on TNF-α-stimulated CCL2 synthesis seems to be mainly receptor-mediated. More importantly, our data are in corroboration with Svensson *et al*. who first reported the measurement of CCL2 in extracellular space in human breast cancer tissues^40^. In our study, human breast tumor sections from 57 patients were stained for CCL2 and Twist using IHC. Approximately 50% of cases showed moderate to strong CCL2 staining despite the tumor subtypes. Our observation, that CCL2 expression was not statistically related to HR status or Her-2 amplification (Table 1), was intriguing since estradiol has been shown to play a key role in CCL2 modulation of ER+ breast cancer cells. Indeed, our findings are supported by previous experimental studies demonstrating the CCL2 levels of breast tumors correlated significantly with serum estrogen levels^40^. Similarly, our data are also in agreement with Ueno *et al*. who reported that expression of CCL2 showed no statistical correlation with ER or PR levels in 135 breast tumor specimens using ELISA analysis. They also demonstrated that CCL2 had significant prognostic values for relapse-free survival, in addition to lymph node status and tumor size^41^, which were both found significantly associated with CCL2 expression in our IHC analysis. Besides, Kim *et al*. showed that serum estradiol level was significantly higher in postmenopausal breast cancer patients with metastasis than those without metastasis, but not statistically related to ER status of breast tumor^42^. And another plausible explanation might be the possibility that more aggressive ER negative breast tumors are associated with higher CCL2 expression since our work also revealed that tumors with high levels of CCL2 staining showed higher aggressiveness of breast cancer.

Our present study also examined the regulatory effects of CCL2 on different breast cancer cells, while disrupting CCL2-CCR2 axis by CCR2 antagonist RS102895 could attenuate these effects partially due to downregulation of MMP-9 and VEGF expression *in vitro*. Thus, our work provided further support that E2-mediated increasing cell proliferation, invasion and angiogenesis are consequences of CCL2 upregulation in ER+ breast cancer cells. In mice implanted with HR+ breast tumors, estradiol also augmented CCL2 levels both in serum and in tumor itself, and blocking CCR2 by RS102895 significantly slowed estrogen-stimulated tumor growth and inhibited liver metastasis largely on account of decreased proliferative activity in tumor cells and reduced angiogenesis and macrophage infiltration in tumor stroma. The tumor modeling results are in agreement with another clinical study which reported that patients with breast cancer had higher CCL2 in serum than healthy controls and this elevated serum CCL2 is closely related to high malignancy and poor prognosis^41^.

Furthermore, our IHC analysis revealed CCL2 was strongly associated with Twist staining (Table 2) and E2 enhanced Twist protein expression in MCF-7 cells in a time-dependent manner (Fig. 4a), indicating that the upregulated Twist expression may be vital to E2-induced CCL2 production. In order to support these data, MCF-7 cells were transfected with different siRNAs specific to silence Twist expression. More importantly, knockdown of Twist significantly inhibited CCL2 secretion in comparison with the controls and even abrogated E2-induced autocrine CCL2 at the indicated time points (Fig. 4c). This experiment provides additional evidence that E2 increases CCL2 expression via Twist upregulation. Hence, further studies focused on determining the mechanism of this Twist upregulation. Differ from a previous research which showed the cytokine induction of CCL2 depended on interaction between AP-1 and NF-κB that binded to the CCL2 promoter region^43^, our data suggest that E2 can indirectly affect CCL2 expression by NF-κB p65 phosphorylation that led to enhancement of Twist nuclear translocation. This process depended on activation of PI3K/AKT signaling instead of ERK1/2 phosphorylation, because E2 didn’t significantly enhance ERK1/2 phosphorylation in either MCF-7 or T47D cells, and disruption of ERK1/2 signaling by MEK inhibitor PD0325901 hardly change the E2-induced NF-κB p65 Ser536 phosphorylation and protein levels of Twist, which implies estrogen-induced CCL2 expression might not mediated by this signaling pathway.

We demonstrate for the first time that autocrine CCL2 is associated with PI3K/AKT/NF-κB pathway and ER signaling between which there may exist cross-talk. On one hand, in the presence of an ER ligand, Twist expression which is essential for autocrine CCL2 as shown by our gene knockdown experiments, was elevated by phosphorylation of AKT on Ser473 (Fig. 5a). Disruption of PI3K/AKT/NF-κB pathway using kinase inhibitors decreased Twist nuclear translocation mediated by E2, indicating this pathway is vital in activating estrogen-induced CCL2 autocrine. Besides, there is no sign of activated AKT phosphorylation or increased Twist expression in ER-negative MDA-MB-231 cells (Fig. 5b), implying a potential effect for ER signaling on E2-triggered PI3K/AKT pathway activation. On the other hand, independently of E2 presence, CCL2 is also capable of phosphorylating AKT on Ser473 via CCR2. Similar to the studies with estrogen, disruption of CCL2-CCR2 axis by RS102895 diminished phosphorylation of AKT on Ser473 and downregulated expression of MMP-9 and VEGF mediated by CCL2 (Fig. 5c), indicating CCR2 is critical for CCL2 in the activation of PI3K/AKT signaling. This is entirely conceivable considering previous evidence pointed that CCR2 activation could result in the key elements phosphorylation in PI3K/AKT pathway^44^.

Previous studies have reported that various intracellular signalings are capable of activating ER without binding with hormones^45,46^. In our experiment, a slight ER phosphorylation on Ser118 (Fig. 5a) instead of Ser167 was shown in both T47D and MCF-7 cells treated with estradiol, which is highly conceivable in consideration of ER binding with estrogen. Notably, ER antagonist tamoxifen did not entirely neutralize the impact of estrogen on CCL2 secretion and AKT phosphorylation to the control levels. More and more evidence has indicated the resistance to estrogen-blocking treatment is associated with upregulated signal transduction pathways of receptor tyrosine kinase, such as EGFR and Her-2, which can lead to a series of gene transcription that might participate in CCL2 production without estrogen binding with ER^47,48^. Similarly, our observations indicate that PI3K/AKT/NF-κB pathway that cannot be totally inhibited by tamoxifen, may contribute to the E2-induced CCL2 autocrine independently of ER activation.

In light of a previous study showing that ER phosphorylation on Ser167 via PI3K/AKT pathway adds the possibility of endocrine therapy resistance^49^, our novel findings shed new light on estrogen-induced autocrine CCL2 via PI3K/AKT/NF-κB signaling, which makes it more conceivable that CCL2-CCR2 axis may get involved in the process of acquired tamoxifen resistance. Thus, blocking CCL2-mediated crosstalk between breast cancer cells and normal endothelial cells could be an approach to improve efficacy of endocrine therapy, especially considering that disruption of CCL2-CCR2 axis was proved to inhibit breast cancer cell proliferation and invasion in our current study. Besides, antagonism therapy against CCR2 strikingly suppressed estrogen-triggered breast cancer growth, vascularization, macrophage infiltration and metastasis in xenograft tumor models, suggesting a critical role of CCL2-CCR2 axis in HR+ tumor progression.

Although we described that E2 can directly induce autocrine CCL2 in ER+ breast cancer, our data shown here suggest the modulatory effect of estrogen on tumor cells and normal endothelial cells cannot be due to the operation of CCL2 alone, and other secreted cytokines might also get involved in this process. Future investigations are needed to determine the levels and functions of additional molecules also recognized in E2-induced CM, and the mechanisms through which they interact with CCL2.

In summary, our observations imply that hormonal regulation of CCL2 is essential to development and progression of HR-positive breast cancer. Estrogen-triggered CCL2 modulation is mainly dependent on HR status and PI3K/AKT/NF-κB signaling activation. Twist serves as a crucial mediator to induce autocrine CCL2. Our current research reveals a previously unrecognized mechanism by which estrogen affects CCL2 regulation in ER+ breast cancer and identifies CCL2-CCR2 axis as a new feasible target for resistance to endocrinotherapy. The profound tumor microenvironment remolded by estrogen and chemokines like CCL2 shed light on new therapeutic regimes for hormone-dependent breast cancer.

## Methods

A whole description of methods and materials, including ELISA, IHC staining and evaluation, qRT-PCR, Western blot, cell viability, migration and invasion assays, tube formation assay and immunofluorescence assay were described in the Supplementary Information File.

### Patients and samples

The experimental protocol was approved by the Human Ethics Committee of the First Affiliated Hospital of Xi’an Jiaotong University and written informed consent was acquired. 57 breast cancer patients (age range, 33–77 years old) who surgically treated without any preoperatively anti-tumor therapies at the Department of Breast Surgery, the First Affiliated Hospital of Xi’an Jiaotong University from 2013 to 2015 were recruited. Tumors from all patients were evaluated by pathologists following surgery and subjected to TNM staging system, according to AJCC (the 7^th^ version, 2010). Their clinicopathological characteristics are summarized in Table 1.

### Cell culture

Human breast cancer MCF-7, T47D, MDA-MB-231 and SK-BR-3 cell lines at passages 3 to 15 were sourced from Shanghai Cell Bank, Chinese Academy of Sciences (Shanghai, China). All cells were maintained in 90% DMEM or Leibovitz’s L15 supplemented with 10% fetal bovine serum (FBS; Gibco, Grand Island, NY, USA) and cultured at 37 °C in a humidified incubator supplemented with 5% carbon dioxide.

### Transient siRNA Transfection

Negative control siRNAs (5′-UUCUCCGAACGUGUCACGUTT-3′, 5′-ACGUGACACGUUCGGAGAATT-3′) and siRNAs targeting Twist (Si-Twist864: 5′-GAUGGCAAGCUGCAGCUAUTT-3′, 5′-AUAGCUGCAGCUUGCCAUCTT-3′; Si-Twist782: 5′-GCAAGAUUCAGACCCUCAATT-3′, 5′-UUGAGGGUCUGAAUCUUGCTT-3′) were synthesized by GenePharm (Shanghai, China). SiRNAs were transfected into cells according to the manufacturer’s protocol. Briefly, MCF-7 cells were seeded at a density of 5 × 10^4^ cells each well in a 24-well plate and cultured for 24 h. Cells were then washed with PBS and incubated in Opti-MEM (Invitrogen) with a complex of 20 pmol of Twist siRNA or control siRNA and 1ul of Lipofectamine 2000 transfection reagent (Invitrogen) for 6 h. The medium containing siRNA-transfection reagent complexes was then aspirated and replaced with DMEM containing 10% FBS for 24 h prior to stimulation with E2.

### Orthotopic tumor xenografts

All procedures were conducted according to the guidelines approved by the Animal Care and Use Committee of Xi’an Jiaotong University. 4-week-old female athymic nude mice were purchased from Silaike Laboratory Animal (Shanghai, China) and housed in a pathogen-free animal facility supplied with autoclaved water and food. 1 × 10^7^ MCF-7 or MDA-MB-231 cells in a 1:1 (v/v) mixture of FBS-free media and Matrigel (BD Biosciences) were injected orthotopically into their left mammary gland (n = 5 per group). Mice were randomly divided into DMSO and E2 group treated with or without RS102895, a small molecule CCR2 antagonist. Estradiol was intraperitoneally (i.p) injected (1.5 mg/kg) to mice every 2 days. RS102895 was also i.p injected (2 mg/kg) every 2 days. Tumors were measured using a vernier caliper and their volume was calculated using the formula: V = d^2^ × D/2, where d = minimal diameter and D = maximum diameter. Then the mice were euthanized, after which their tumors and livers were dissected and the serum was collected. Serum concentration of CCL2 was measured using a mouse CCL2 ELISA kit (eBioscience). The metastatic tumor nodules on livers were counted and showed by H&E staining. The tissue sections were stained by IHC using anti-CCL2 (R&D Systems), anti-Twist (Abcam), anti-PCNA (Santa Cruz Biotechnology) and anti-CD31 (Abcam).

### Statistical analyses

Data from experiments performed in triplicate at the minimum are presented as means ± SEM. The two-tailed Student’s t test and ANOVA with Bonferroni’s post-test of comparisons were used with GraphPad Prism 5.0 (GraphPad Software, Inc., CA, USA). The relationship between CCL2 expression and clinicopathologic characteristics were analyzed with a two-tailed Chi-square test or Fisher’s exact test using the SPSS software (version 18.0; SPSS, Inc., Chicago, IL, USA). The correlation between CCL2 and Twist expression was analyzed with Spearman’s rank test. *P* < 0.05 was considered statistically significant.

### Data availability

All data generated or analyzed during this study are included in this published article and its supplementary information files.

### Ethics approval and Informed consent

All procedures performed in this study involving human breast cancer specimens were approved by the Human Ethics Committee of the First Affiliated Hospital of Medical School of Xi’an Jiaotong University and were in accordance with the ethical standards. Written informed consent was obtained from all individual patients. All animal experiments were performed in accordance with the ethical standards of the Animal Care and Use Committee guidelines of Xi’an Jiaotong University, Shaanxi, China.

## Electronic supplementary material

Supplementary Information File
