# Supplementary material for: Estrogen promotes progression of hormone-dependent breast cancer through CCL2-CCR2 axis by upregulation of Twist via PI3K/AKT/NF-κB signaling
Source: Sci Rep. 2018 Jun 22;8:9575. doi: 10.1038/s41598-018-27810-6 (PMC6015029; doi:10.1038/s41598-018-27810-6)
Supplement: Supplementary file 1 — Supplementary Information File [file 41598_2018_27810_MOESM1_ESM.pdf]

Estrogen promotes progression of hormone-dependent breast cancer through CCL2-CCR2 axis by upregulation of Twist via PI3K/AKT/NF-κB signaling

Rui Han [1], Shanzhi Gu [2], Yujiao Zhang [1], Anqi Luo [1], Xin Jing [1], Lin Zhao [1], Xinhan Zhao [1,*], Lingxiao Zhang [1,**]

[1] Department of Oncology, the First Affiliated Hospital of Medical School of Xi'an Jiaotong University, 277 West Yanta Road, Xi'an, Shaanxi Province, 710061, China

[2] Department of Forensic Medicine, Medical School of Xi'an Jiaotong University, 76 West Yanta Road, Xi'an, Shaanxi Province, 710061, China

Supplementary Methods

ELISA analysis

4×10^5 breast cancer cells/well were seeded in duplicate into 24-well plates, and incubated in 500 μl/well complete media for 24 h. Then cells were treated with 0.1% (v/v) dimethyl sulphoxide (DMSO), 10nM 17β-estradiol (E2; Sigma-Aldrich, St Louis, MO, USA) or 0.5μM tamoxifen (TAM; Sigma-Aldrich) plus 10nM E2 which were added in serum-free DMEM or Leibovitz's L15 and incubated for 24 h, 48 h or 72 h. The supernatant was collected and the culture media from cells incubated for 48 h was used as conditioned media (CM) for the indicated experiments. The human CCL2 ELISA kit (eBioscience, San Diego, CA, USA) was used to measure CCL2 concentration, following the manufacturer's instructions.

Immunohistochemistry staining

CCL2 and Twist staining were performed on array slides containing sections of paraffin-embedded, formalin-fixed tumor tissues. The sections (4μm thickness) were dewaxed, rehydrated in a series of graded ethanol solutions and then subjected to microwave radiation as antigen retrieval in sodium citrate buffer, pH 6.0 for 15 min. Endogenous peroxidase activity was blocked with 3% H_2O_2 in methanol for 15 min at room temperature. The sections were washed three times with PBS and blocked in PBS containing 5% goat serum for 30 min at room temperature. Next, the sections were incubated with antibodies to CCL2 (R&D Systems, USA) and Twist (Abcam, Cambridge, MA, USA), overnight at 4 °C. Samples were warmed to room temperature, then washed three times with PBS, incubated with secondary goat biotinylated antibodies for 30 min at room temperature, conjugated with streptavidin peroxidase, and washed again, after which immune complexes were detected with 3,3′-diaminobenzidine substrate (DAB, DAKO). Sections were counterstained with Mayer's hematoxylin for 5 min, dehydrated, and mounted with Cytoseal. For analysis, the stained samples were evaluated microscopically (at 200× and 400× magnification) by two independent pathologists in a blinded manner. Brown cytoplasmic staining for CCL2 and brown nuclear or cytoplasmic staining for Twist were considered positive. Based on the sum of staining intensity and the percentage of positively stained tumor cells, the staining for CCL2 and Twist in tumor cells was scored as 0 (negative/no staining), 1+ (weak staining), 2+ (moderate staining) and 3+ (strong staining). For F4/80 quantification, five random high-power fields (HPFs) at 200× magnification in F4/80 positive hot spots were scored on a scale of 0–6 for staining intensity and distribution within a field: 0, undetectable; 1, faint, discrete

patches; 2 faint, all over; 3 medium, discrete patches; 4 medium, all over; 5 intense, discrete patches; 6 intense, all over. Negative controls were also evaluated for each IHC marker.

RNA extraction and quantitative real-time PCR (qRT-PCR)

Total RNA was extracted from different breast cancer cells using TRIzol™ reagents (Invitrogen, CA, USA). RNA (1 μg) was used for cDNA synthesis by reverse transcription using a PrimeScript RT reagent kit (TaKaRa, Dalian, China). The real-time PCR was carried out using a SYBR Green PCR Kit (TaKaRa), according to the manufacturer's instructions. GAPDH expression was assessed as an internal control to standardize the expression level of target gene. The comparative expression level of target gene = $2^{-\Delta\Delta CT}$. The sequences of the primers for real-time PCR are listed in Supplementary Table S1.

Western blot assay and antibodies

Breast cancer cells were seeded in 6-cm dishes at a density of 2×10^5 cells, cultured for 24 h, and starved in serum-free media for 6 h. Cells were then treated with serum-free media containing 10nM E2 or 0.1% (v/v) DMSO at 37 °C in the presence or absence of 0.5μM tamoxifen, 50μM LY294002 (Selleck Chemicals, Houston, TX, USA), 10μM BAY11-7082 (Selleck Chemicals) or 20μM PD 0325901 (Selleck Chemicals). MCF-7 cells were also treated with 20 ng/ml rhCCL2 in the presence or absence of 20μM RS102895. Then the cells were harvested, and total protein was extracted from the stable cell lines. Equal amounts of protein (150μg) were separated by sodium dodecyl sulfate-polyacrylamide gel electrophoresis (10% SDS-PAGE) and then transferred onto a polyvinylidene difluoride (PVDF) membrane (Roche). The immunoblots were incubated in 5% (w/v) BSA dissolved in TBST (10 mM Tris–HCl, pH 8.0, 150 mM NaCl, and 0.05% Tween-20) for 2 h at room temperature. Next, the blots were probed first with specific antibodies and then with the appropriate secondary antibodies. β-actin was used as a loading control.

Antibodies were purchased from the following sources: anti-Twist antibody (Abcam, Cambridge, MA, USA), anti-MMP-9 antibody (Proteintech Group Inc., USA), anti-VEGF antibody (Abcam), anti-AKT antibody (Cell Signaling Technology, Boston, MA, USA), anti-NF-κB p65 antibody (Cell Signaling Technology), anti-ERK1/2 antibody (Cell Signaling Technology), anti-ERα antibody (Cell Signaling Technology), anti-β-actin antibody (Santa Cruz Biotechnology, Santa Cruz, CA, USA).

Cell viability assay

Cells in the exponential phase of growth were harvested and seeded into 96-well plates at a density of 3,000 cells per well in DMEM or Leibovitz's L15 with 10% FBS and cultured at 37°C and 5% CO2 humidified incubator for 24h. Then the medium was removed and replaced with 100 μl DMEM or Leibovitz's L15 containing rhCCL2 (PeproTech, Rocky Hill, NJ, USA) at the concentration of 0, 25, 50, 100 and 200 ng/ml, or CM from cells treated with tamoxifen and E2 or E2 alone. For blocking experiment, RS102895 (20μM; Tocris, Bristol, UK) was added to the cells 2h before the addition of rhCCL2. WST-8 cell viability assay (Cayman Chemical, Ann Arbor, MI, USA) were performed 24, 48 and 72 h after treatment. Briefly, 10 μl/well WST-8 was added to each well and incubated for 1 h. The absorbance was measured at 450 nm using an automated microplate reader Model 680 (Bio-Rad Laboratories, West Berkeley, CA, USA).

Cell migration and invasion assays

The migration assay was conducted via transwell experiments. In brief, 5×10^4 breast cancer cells were trypsinized, washed, and resuspended in serum-free medium and seeded in the upper well of a 8μm pore-size 24-well transwell plate (Millipore Co., Billerica, MA, USA). 500μl of DMEM or Leibovitz's L15 with 10% FBS culture media containing a serial dilution of rhCCL2 or CM from cells with or without treatment of E2 was added to the bottom well as a chemoattractant. CCR2 antagonist RS102895 (20μM) was added to the cells 2h before they were planted in the upper compartment. After 24 hours of incubation, the non-migrated cells were then carefully removed from the upper surface (inside) of the well with a wet cotton swab. Cells that had migrated to the lower surface of the membrane were fixed for 15 min in 4% formaldehyde and stained with 0.01% crystal violet solution for 30 minutes. The numbers of cells that migrated were determined from five random HPFs visualized at 100× magnification, and means were obtained for statistical analysis.

The invasion assays were performed in a similar manner as the migration assays, except that 1×10^5 cells were placed in the upper compartment evenly coated with a layer of Matrigel (BD Biosciences, San Jose, CA, USA) which was allowed to polymerize at 37°C for 2h and incubated for 36 h. The numbers of invading cells were also quantified from five random HPFs visualized at 100× magnification.

Tube formation assay

Stable MCF-7 and T47D cells were incubated at 37˚C with serum-free media in the presence or

absence of 10nM E2 for 48 h. Wells of a 24-well plate were coated with 100 μl of Growth Factor Reduced (GFR) Matrigel (BD Biosciences) and incubated at 37˚C for 60 minutes. Human Umbilical Vein Endothelial Cells (HUVECs; 2×10^4 cells/well) were suspended in 500 μl/well of the CM under study and plated on the Matrigel. Plates were incubated at 37˚C in 5% CO_2 with humidity for indicated time. After incubation, imaging of five representative fields per well was performed using phase contrast microscopy (at 100× magnification). The number of branches was counted using Image J software (National Institutes of Health, USA).

Immunofluorescence assay

A total of 2 × 10^4 cells per well were grown on glass coverslips in a 24-well plate overnight. When the cells were 60–70% confluent , they were washed twice with PBS, then fixed in 4% paraformaldehyde solution, and permeabilized in 0.05% Triton X-100 (Sigma) in PBS for 15 min. The cells were then washed three times (5 min each time) with PBS and blocked with 5% BSA in PBS for 1 h at room temperature. The cells on the coverslips were incubated in a humidified box with the respective primary antibodies at a 1:100 dilution overnight at 4°C. Next, the cells were washed three times (5 min each time) in PBS and incubated for 1 h with dylight 549-conjugated secondary antibodies at a 1:100 dilution (Abbkine, CA, USA) at room temperature in the dark. Finally, the cells were washed three times with PBS and incubated with 1μg/ml 4, 6-diamidino-2-phenylindole (DAPI, Roche) for 5 min at room temperature in the dark. Then, the slides were washed extensively with PBS and observed with an immunofluorescence microscope (Nikon, Japan) with identical exposure times at 100× and 200× magnification.

Supplementary Table S1. Primers for quantitative real-time PCR

Name	Primer sense	Primer sequence (5’–3’)
GAPDH	Forward	GCACCGTCAAGGCTGAGAAC
	Reverse	TCCACCACCCTGTTGCTGTA
CCL2	Forward	CTTCTGTGCCTGCTGCTCATA

	Reverse	CTTTGGGACACTTGCTGCTG
MMP-9	Forward	CCCTGGAGACCTGAGAACCAA
	Reverse	CATCTCTGCCACCCGAGTGTA
VEGF	Forward	CATCCAATCGAGACCCTGGTG
	Reverse	TTGGTGAGGTTTGATCCGCATA
Twist	Forward	CAGCTACGCCTTCTCGGTCT
	Reverse	ACTGTCCATTTTCTCCTTCTCTGG

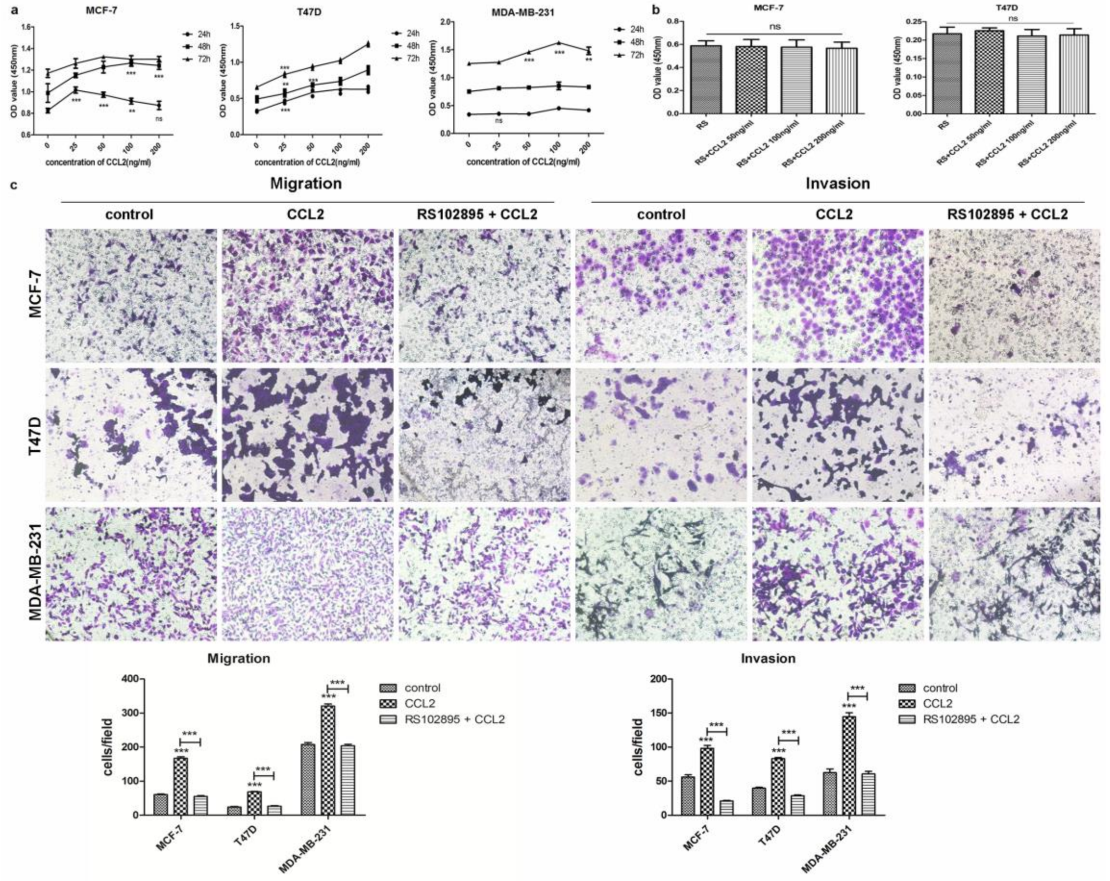

Supplementary Figure S1. CCL2-CCR2 axis coordinates breast cancer cell proliferation, migration and invasion *in vitro*. **(a)** MCF-7, T47D and MDA-MB-231 cells were treated with 0-200 ng/ml CCL2 for 24h, 48h or 72h and then subjected to WST-8 cell viability assay; **(b)** MCF-7 and T47D cells were treated with 0-200 ng/ml CCL2 for 48h in the presence of CCR2 antagonist RS102895 (20μM) and then measured for cell viability by WST-8 assay; **(c)** MCF-7, T47D and MDA-MB-231 cells were treated with or without 50ng/ml rhCCL2 in the pretreatment or absence of RS102895 (20μM). Then their migratory ability at 24 h and invasive ability at 36 h post culture were respectively determined by

transwell migration and invasion assay. Data are expressed as the mean ± SEM of each group of cells at each time point from three separate experiments. (** P<0.01, *** P<0.001 vs control or DMSO CM; ns, not significant)

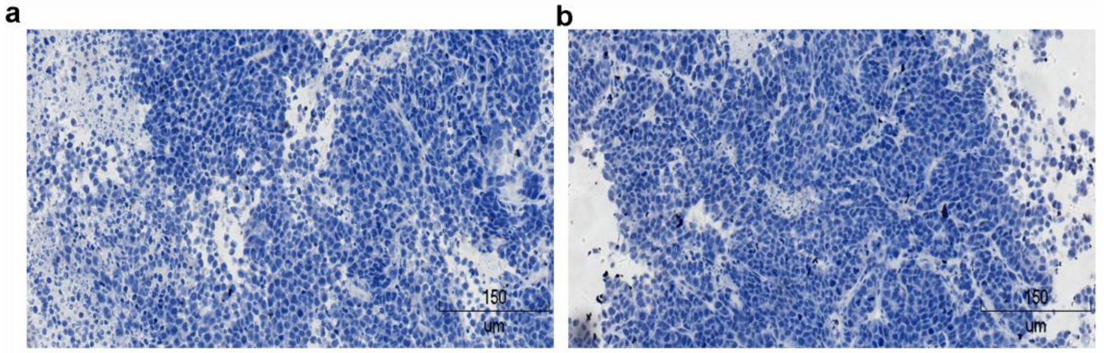

Supplementary Figure S2. Images of mouse tissue sections stained for CCL2 and Twist. **(a)** Negative control for anti-CCL2 staining; **(b)** Negative control for anti-Twist staining. Scale bar, 150 μm.

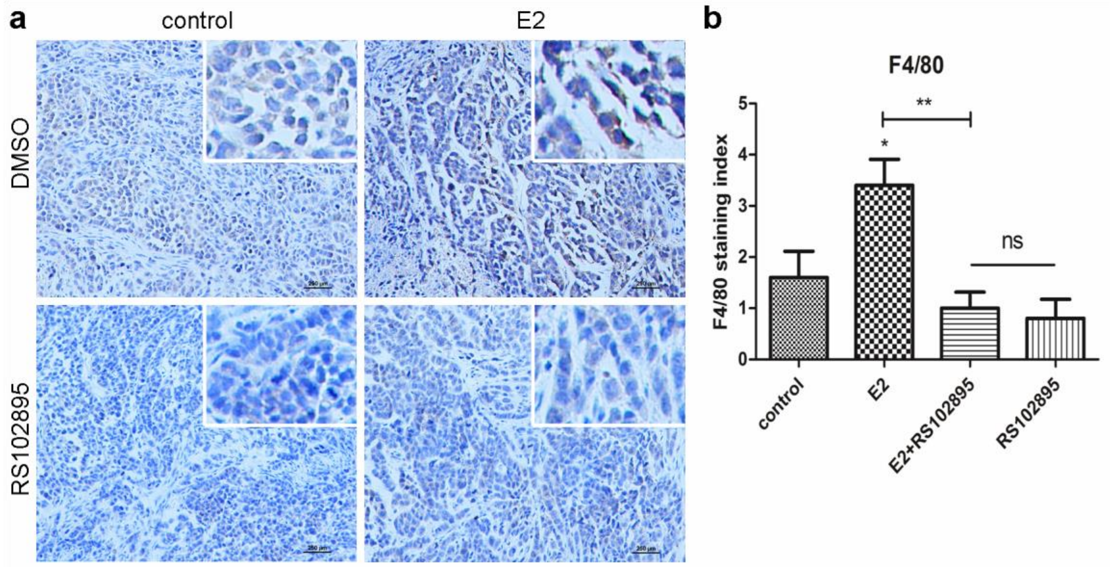

Supplementary Figure S3. E2 promotes macrophage infiltration via CCL2-CCR2 axis in xenograft tumor models. **(a)** Images of implanted tumor tissue sections stained for mature macrophage marker F4/80. Insets show magnification for staining detail. Scale bar, 250 μm. **(b)** Quantification analysis of anti-F4/80 staining. Five random high-power fields per section were scored for anti-F4/80 staining intensity and distribution. Data are expressed as the mean ± SEM of different sections from each group of mice. (* P<0.05, ** P<0.01 vs control or RS102895/E2+RS102895; ns, not significant)

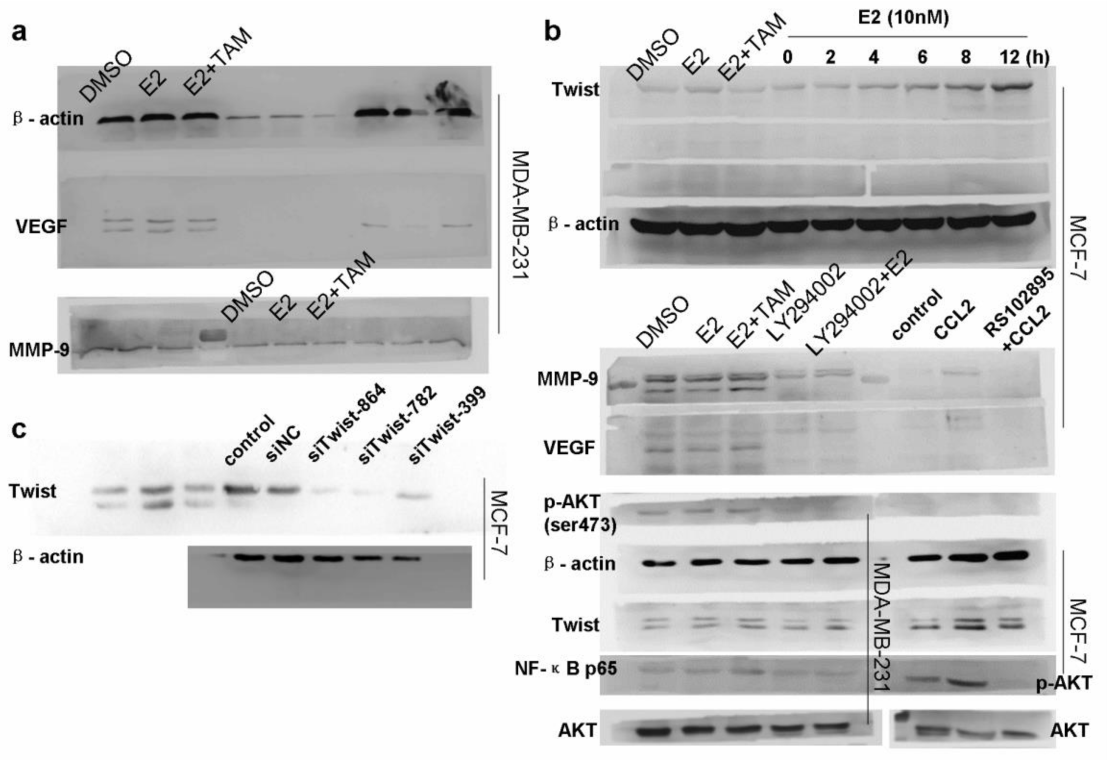

Supplementary Figure S4. Full-length breast cancer cell western blots for cropped gels/blots. **(a)** Total cell extracts of MDA-MB-231 cells with indicated treatments were probed for VEGF and MMP-9. β-actin was loading control. Samples and antibodies were processed and run on different gels due to the similar molecular weight of VEGF and β-actin; **(b)** Samples from MCF-7 cells with indicated treatments were detected for Twist and β-actin on the same gel as well as VEGF and MMP-9. MDA-MB-231 cells were also stimulated with 10nM E2 in the presence or absence of tamoxifen and LY294002, followed by probing for AKT phosphorylation and Twist on the same gel, meanwhile another gel was also used to examine NF-κB p65 expression; **(c)** MCF-7 cells were transfected with different siRNAs then probed for Twist to examine the knockdown efficiency. These five samples were processed and run on the same gel.
